# Supplementary material for: Treating rheumatoid arthritis in Zanzibar: a cost effectiveness study comparing conventional, biologic, and targeted-synthetic disease modifying anti-rheumatic drugs
Source: Front Med (Lausanne). 2025 Aug 20;12:1618493. doi: 10.3389/fmed.2025.1618493 (PMC12405486; doi:10.3389/fmed.2025.1618493)
Supplement: Supplementary file 1 [file Data_Sheet_1.docx]

Supplementary 1: Markov model


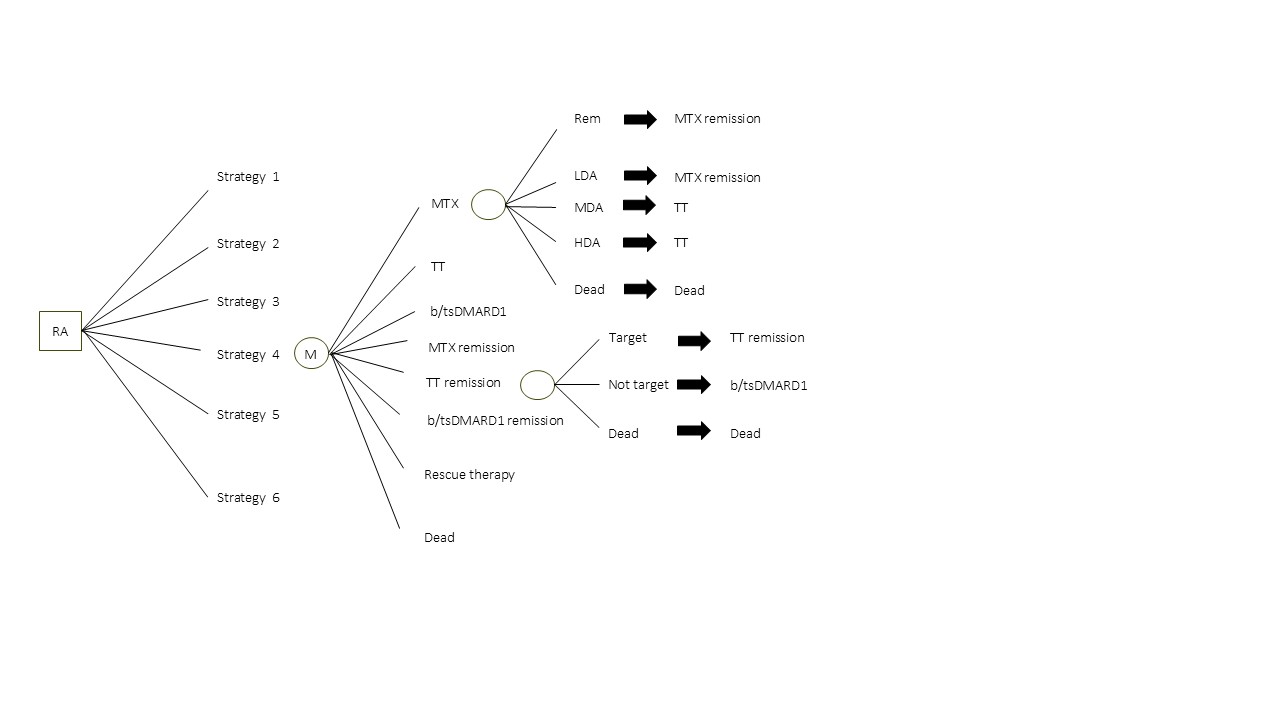


Supplementary 2: Capital costs

The clinic caters to about 13,000 patients per year and costs were estimated per patient equating to USD 2.4, 0.06 and 0.23 for the building, supplies and electricity. Only outpatient clinic visits were considered. It was assumed that the doctor, nurse and pharmacist would each require 20, 10 and 5 minutes per patient respectively. The costs equated to USD 1.24, 0.2 and 0.13 per patient per clinic visit for doctor, nurse and pharmacist respectively. No considerations were made for the management of side effects or in-patient care.

Supplementary 3: Price threshold analysis results

| **Cost of TT at baseline** | **ICER at baseline** | **WTP threshold** | **Calculation count** | **Threshold cost** |
| --- | --- | --- | --- | --- |
| 283 | 5136.12 | 282 | 4 | 101.77 |
|  |  | 1136 | 38 | 133.65 |
|  |  | 2272 | 4 | 176.07 |
| **Cost of b/tsDMARD1 at baseline** | **ICER at baseline** | **WTP threshold** | **Calculation count** | **Threshold cost** |
| 94 | 1030.18 | 282 | 4 | 61.54 |
|  |  | 1136 | 5 | 98.59 |
|  |  | 2272 | 8 | 147.88 |
| **Cost of** **b/tsDMARD2 at baseline** | **ICER at baseline** | **WTP threshold** | **Calculation count** | **Threshold cost** |
| 5456 | 32411.35 | 282 | 3 | No threshold found |
|  |  | 1136 | 7 | 111.49 |
|  |  | 2272 | 4 | 305.61 |
